# The status of depression and anxiety in infertile Turkish couples

**Published:** 2011

**Authors:** Mert Kazandi, Ozlem Gunday, Timucin Kurtulus Mermer, Nuray Erturk, Erdinc Ozkınay

**Affiliations:** Department of Obstetrics and Gynecology, Ege University, İzmir, Turkey.

**Keywords:** *Depression*, *Anxiety*, *Infertility*

## Abstract

**Background:** Infertility is a major psychosocial crisis as well as being a medical problem. The factors that predict psychosocial consequences of infertility may vary in different gender and different infertile populations.

**Objective:** The primary purpose of this study was to investigate whether Turkish infertile couples had higher levels of depression and anxiety when compared to non-infertile couples. Our secondary aim was to evaluate the relationship between sociodemographic characteristics and levels of depression and anxiety in Turkish infertile couples.

**Materials and Methods:** We designed a descriptive cross sectional study of 248 infertile women and 96 infertile men with no psychiatric disturbance and 51 women and 40 men who have children to evaluate the depression and anxiety levels between infertile couples and fertile couples. A gynecologist evaluated participants for demographic data and then they were visited by a psychologist to perform questionnaire scales which were The Beck Depression Inventory and the State-Trait Anxiety Inventory for the evaluation of the degree of psychopathology. The data were statistically analyzed, with p<0.05 as the level of statistical significance.

**Results:** We observed significant differences between the infertile couples and fertile couples with respect to state and trait anxiety (p<0.0001) while no difference was regarding with depression, both of women and men. Anxiety and depression were observed as independent from gender when infertile women and men were compared (p=0.213).

**Conclusion:** We believed that the psychological management at infertile couples must be individualized with cultural, religious, and class related aspects.

## Introduction

Infertility that affects almost entire society is a crisis with cultural, religious, and class related aspects, which coexists with medical, psychiatric, psychological, and social problems. It has been defined as the failure to conceive after a year of regular intercourse without contraception and affected 10-15% of couples of reproductive 

age (1). Psychological problems coexisted with infertility were associated with some serious medical conditions and poor treatment outcomes (2, 3). Infertility has also psychological and social aspects, as well as being a medical problem. Many reports in literature indicated that levels of depression and anxiety are high in infertile women (4-10). On the other hand, there are some studies that show that there is no statistically significant difference between the depression and anxiety levels of both fertile and infertile group (11-13).

For the last 15 years, the reports according to psychopathology and infertility indicated two theoretical models that infertility was a reason or a consequence. The first model, which is rejected by many authors, indicates the role of psychogenic factors as a reason of infertility (14) although this hypothesis was indicated by some evidence (15). The second model emphasizes that psychological and social stress is secondary to infertility (16, 17).

For many couples, infertility causes several serious social and psychological consequences such as, personal distress, reduced self-esteem and loss of correct partner relationship. These results differ in different cultures (18)**.** For example, Turkish women often come face to face with questions such as, “Are you married? Do you have a child?”. Moreover, some Turkish people, mostly the former generations, still believe that infertility is exclusively limited to female causes. Thus, women labeled as infertile feel disgrace and fault leading to anxiety and depression. In developing countries, economic privation can arise psychological distress because many families especially in old age depend on children for sustentation (19). Besides, some infertile couples are forced to take care of their parents. Same situation can develop in the future for them. 

The reports regarding with infertility that associated anxiety and depression were published mostly in developed countries. Most of the studies from developing world indicated to social aspects of infertility. Moreover, a few of them provided quantitative data related to psychological disorders coexisted with infertility (20-31). Additionally, infertile women were investigated more than infertile men by these studies (21, 22, 26-30). It was reported that there is high rate of depression among infertile couples in Southern Iran (20). In the same way, a study in Kuwait shows infertile women exhibited significant higher levels of anxiety and depression, because childlessness results in social stigmatization for infertile women (21). Similar findings were found for infertile women in a Turkish study (22). However, the different outcomes were reported regarding with relationship between psychosomatic disorders and related factors in infertile couples at most of them. This study was designed to assess whether Turkish people who were suffering from couple infertility had higher levels of depression and anxiety when compared to non-infertile controls. Our secondary aim was to evaluate relationship between sociodemographic characteristics and levels of depression and anxiety in Turkish infertile couples.

## Materials and methods

This is a a descriptive cross sectional study conducted in a total of 248 women and 96 men with no psychiatric disturbance who were referred to Infertility Department of Ege University Medicine Faculty from March 2004 to January 2007 for treatment of their infertility problems. Inclusion criteria were diagnosis lasting a minimum of 3 months due to infertility disorders, failure to conceive despite regular sexual intercourse (4-5 times per week) sustained for a period exceeding 12 months, no contraception in the last 12 months, inability to conceive and lack of pregnancy in patient history (primary infertility). The fertile group consisted of healthy 51 women and 40 men. This group was selected from all available couples who attempting outpatient gynecological clinics of our hospital for control between the ages of 18 and 45 years, married, Turkish, having at least one child and absence of current clinical psychiatric disorders. We were dealt to equalize the infertile couples’ and fertile couples’ sample size because of reaching statistical adequate minimal fertile group sample size according to our primary aim of study. Then, we raised infertile sample size for our secondary aim.The subjects were informed about the aims of the study and written permission was given. A gynecologist evaluated the participants for demographic data (age, marriage duration, education, occupation, medical history, health problems, gynecological history, infertility duration and diagnosis) regarding the study and then they were visited by a psychologist to perform questionnaire scales which are The Beck Depression Inventory (BDI) (32) and the State-Trait Anxiety Inventory (STAI-S/T) (33) for the evaluation of the degree of psychopathology. 

We used The Turkish version of the BDI with a satisfactory validity and internal consistency (Cronbach’s alpha:0.80) (34), a 21-item test, to assess the severity of depressive symptom. It is useable both in specific clinical populations (psychiatric) and non-specific (general population) for this purpose. Each item describes a specific behavioral manifestation of depression. 

A valid and reliable Turkish version of STAI-S and STAI-T (35) is another component of the research questionnaire, recommended for the evaluation of anxiety occurrence (Cronbach’s alpha: 0.92 and 0.86, respectively). It is divided into two sections each consisting of 20 items. The first 20 items measure state anxiety (STAI-S) and the second 20 items measure trait anxiety (STAI-T). It clearly differentiates between the temporary condition of “state anxiety” and the more general and long-standing quality of “trait anxiety”. Frequency tables were formed for variables. 

**Statistical analysis**

Comparisons between the groups were made using Chi-square test and the Mann–Whitney U test, with p<0.05 as the level of statistical significance. 

## Results

There were significant differences between the infertile couples and fertile couples with respect to state and trait anxiety while there were no differences regarding with depression, both of women and men (p<0.0001). Anxiety and depression were observed as independent from gender when infertile women and men were compared.

At infertile women, the sociodemographic characteristics were compared with anxiety and depression rates. Depression ratios in infertile housewives were higher (p<0.021) but state and trait anxiety did not differ according to working. High school graduation was related to state and trait anxiety with regard to education levels (p<0.03 and p<0.003, respectively), while it was not correlated with depression. There were no significant differences with respect to infertility reason for ratios of anxiety and depression. İnfertility duration was related to depression and the ratios of depression rised with prolonged infertility period (p<0.002). However, state and trait anxiety were not associated with infertility duration. The rates of anxiety and depression for infertile men were also compared with sociodemographic characteristics. No statistically significant differences were detected regarding the age, marriage duration, education, occupation and infertility duration, both for anxiety and depression. Endometriozis as infertility reason was related to only depression.

## Discussion

In this study, we compared the levels of anxiety and depression between infertile and fertile patients. We also observed the relation between the distress and different variables experienced by infertile couples.

Infertility can be defined as a crisis, which coexists with medical, psychiatric, psychological, and social problems. Consequences of infertility arise from short and long-term devastating effects on both individual’s physical and mental health, and marital system (36). Then, infertilty and its psychosocial outcomes were investigated with many studies. The outcomes of them could be effected with many various factors as marital relationship, family support, traditional and social belief, which they were perceived individually. Besides, subjective datas were dependent that questionnaire scalas were filled by patients. The differences between tests, which were used at various studies must be considered during evaluation. Most of the standard tests utilized have limitations in the setting of infertility and the ‘ideal test’ has yet to be designed (37). 

Distress, anxiety and depression are general consequences of infertility. Many studies indicated that incidance of major depression is higher at infertile couples than fertile couples and it ranges 15-54% (38). A similar pattern was observed regarding to anxiety and it was reported that significant anxiety levels were seen at 8-28% of infertile couples (39). Wilson *et al* reviewed previous thirty published reports related to psychological disorders and infertility. Significantly higher psychological distress of patients who referred to an infertility clinic than the control group have been emphasized by them. Same researches indicated that poor long-term well being and higher levels of anxiety and depression were shown between infertile women (39). In our study, the levels of anxiety of infertile women are higher significantly than fertile women, while there is no difference according to levels of depression. It is found that the levels of anxiety are also higher as similar to infertile women at infertile men than fertile men. 

Greil *et al* reported that infertility causes a higher psychological distress level at women than men (41). Infertile women compared with infertile men have significantly higher trait anxiety levels in this study. Meaning of life and marriage for many women can be child bearing. Moreover, the most of diagnosing methods and infertility therapy are performed to woman’s reproductive system, and as an outcome of such therapy, her life may be more annoying than her husband. Women may carry on suspecting themselves, even if their partners are definitely infertile (42). However, men show reactions to infertility by implication. If they are the source of infertility, their responce can be like women. The deficiency of feelings definition and loss of emotion occur for both couples (43). 

There were a few studies regarding with the effect of a gender-specific infertility diagnosis on the couples. The more psychiatric distress among men with male factor infertility compared to men in couples receiving other diagnoses were reported in developed western countries (44-46). However, a study in Taiwan comparing the differences in responses from husbands and wives based on an infertility diagnosis, reported that husbands, regardless of the diagnosis, showed no difference in psychological responses (25). We didn’t find significant relationship between the psychiatric distress and gender-specific infertility reason. It was not surprising that the children carry on the family name and are a consolidation for the future in Turkish culture. Then, the basic origin of psychosocial disorders are motives of wanting children more than the reason of infertility for our population. At infertile men in our study, the levels of depression were associated with the reason of infertility while there is no relation for women. Most of the etiological factors were endometriosis both for women. Endometriosis contributes to distress as a result of reducing life quality by causing dysparonia and chronic pelvic pain and affecting sexual life. 

Ramezanzadeh *et al* reported a study consisting of 370 infertile patients that the relation between psychological disorders and duration of infertility was investigated. They emphasized that anxiety and depression were associated with infertility duration. The levels of anxiety and depression rised as parallel with infertility period (47). However, some studies showed that there is no relation between duration of infertility and depression or psychological factors (48). The findings of another study in Turkey and our study indicate similar consequences but there is an opposite idea regarding with this relationship by Domar *et al* (22, 49). They reported that the levels of depression of women who have three years of infertility history are higher than women who have one year or six years of infertility history (49). This “U” shaped pattern depended on hopeful effects of infertility treatment at the beginning by them. Somehow, the women may get used to this situation after six years and may avoid depression. It’s possible that this “U” shaped pattern will be observed after nine years in our population and may delay acceptance process. Domar *et al* also reported that there is no relation between age and depression levels as similar to our study (49).

In our study, highest depression levels are observed among women who graduated from high school. We think that it may be because of disability of managing stress and problem, although they are aware of the matter. The women with tertiary education may manage their problems easily and the women who graduated from junior school may be protected because of lower levels of awareness. Furthermore, absence from work may rise psychological consequence of anxiety and depression by more tending to problem (50). Additionally in our country, the infertile women with economic hardship have future fear that there is not a child to take care of.

Van Rooij *et al* reported that the levels of emotional distress among infertile Turkish migrants and infertile Turkish people (particularly among women) were high, as compared to those of infertile Dutch (51). In this aspect, we think that sociocultural characteristics are more important than the living place regarding with the source of psychological disorders.

There is a subgroup in infertile people that they require professional psychological assistance. The gynaecologists must consider that psychosocial problems could be accompanied with infertility. It was reported that the reduction of anxiety and depression in infertile couples may rise treatment satisfaction and pregnancy ratios in selected cases (52). Besides, the psychosomatic disorders can develop by infertility therapy in women (53). Nevertheless, the controlling of basal distress is more important than stress related to infertility treatment. If distress is chronic, it will be an affected outcome of therapy (54).

## Conclusion

Infertility can be defined as a crisis, which coexists with medical, psychiatric, psychological, and social problems. Then, there are many studies regarding with infertility and its psychosocial effects. 

The discrepancies between psychosomatic disorders and related factors seem to result from individual reasons as with cultural, religious, and class related aspects. Additionally, the limitations and differences of the standard tests utilized could be contributed to interpretational variety. We believed that the psychological management at infertile couples must be individualized with cultural, religious, and class related aspects.
