# Corrigendum to “The status of depression and anxiety in infertile Turkish couples” [Iran J Reprod Med 2011; 9: 99-104]

**DOI:** 10.18502/ijrm.v21i8.14023

**Published:** 2023-09-20

**Authors:** Mert Kazandi, Ozlem Gunday, Timucin Kurtulus Mermer, Nuray Erturk, Erdinc Ozkınay

The publisher has been informed of an error that occurred on page 99 in which the second authors name must be changed to Ozlem Kayacik Gunday. On behalf of the author, the publisher wishes to apologize for this error. The online version of article has been updated on 31 August 2023 and can be found at https://doi.org/10.18502/ijrm.v9i2.104.

